# Molecular symphonies and signalling pathways orchestrated by the gut microbiome in metastatic colorectal cancer (mCRC); a state-of-the-art review

**DOI:** 10.1017/erm.2024.31

**Published:** 2024-10-28

**Authors:** Nayeralsadat Fatemi, Mahtab Jahdkaran, Seyedeh Nasim Mirbahari, Hamidreza Houri

**Affiliations:** 1 Basic and Molecular Epidemiology of Gastrointestinal Disorders Research Center, Research Institute for Gastroenterology and Liver Diseases, Shahid Beheshti University of Medical Sciences, Tehran, Iran; 2 Celiac Disease and Gluten Related Disorders Research Center, Research Institute for Gastroenterology and Liver Diseases, Shahid Beheshti University of Medical Sciences, Tehran, Iran; 3 Faculty of Sciences and Advanced Technologies in Biology, University of Science and Culture, Academic Center for Education, Culture and Research (ACECR), Tehran, Iran; 4 Department of Genetics, Reproductive Biomedicine Research Center, Royan Institute for Reproductive Biomedicine, Academic Center for Education, Culture and Research (ACECR), Tehran, Iran; 5 Foodborne and Waterborne Diseases Research Center, Research Institute for Gastroenterology and Liver Diseases, Shahid Beheshti University of Medical Sciences, Tehran, Iran

**Keywords:** gut microbiome, metastasis, metastatic colorectal cancer, oncobiosis

## Abstract

The role of oncobiosis, characterized by dysregulated microbial ecosystems associated with cancer, has been increasingly acknowledged in promoting the metastasis and dissemination of tumour cells. A comprehensive understanding of the complex interactions between the gut microbiome and metastatic colorectal cancer (mCRC) presents promising avenues for the development of innovative therapeutic approaches centred around modulating the gut microbiome to prevent or hinder metastatic spread. In this comprehensive review, we aim to provide a molecularly focused summary of the implications of the human gut microbiome and microbial metabolites in the initiation and advancement of mCRC. By elucidating these intricate mechanisms, we strive to establish a foundation for future research and the design of novel interventions targeting the gut microbiome to combat this devastating disease.

## Graphical abstract

**Figure:**
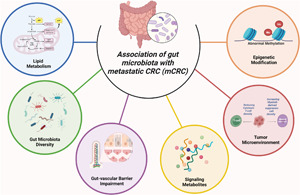
The microbiome's interactions with immune cells, modulation of gene expression, and involvement in DNA damage response highlight its critical role in cancer progression and metastasis, offering potential therapeutic targets for intervention.

## Introduction

Colorectal cancer (CRC) stands as the second most prevalent cause of cancer-related mortality, with an annual diagnosis of nearly two million new cases (Ref. 1). The metastatic spread of cancer cells is the foremost contributor to fatalities in CRC patients, with the liver, lungs, bones and brain being frequent sites of dissemination. Liver metastasis affects approximately half of CRC patients, while 15–23% of patients are diagnosed with metastases at the time of initial assessment (Ref. 2). The aetiology of CRC is believed to stem from a complex interplay of genetic and environmental factors, inclusive of disruptions in the gut microbiome, unhealthy dietary patterns prevalent in the Western world, smoking, obesity, excessive alcohol consumption and chronic colitis associated with inflammatory bowel disease (IBD) (Ref. 3). In addition to established risk factors and genetic predispositions, recent investigations have illuminated the intricate relationship between the gut microbiome and CRC, advancing the compelling proposition that the complex intestinal ecosystem significantly influences the onset and progression of this formidable malady (Ref. 4).

‘Dysbiosis’, an imbalance in the gut microbiome, has been implicated in numerous adverse effects, such as heightened inflammation, compromised immune function and disrupted metabolism. This dysregulation is thought to contribute to the development and aggravation of a spectrum of disease pathologies, including various immune-mediated conditions such as IBD, gastrointestinal (GI) cancers and even neurodegenerative ailments. These outcomes may foster an environment conducive to the proliferation and progression of cancer cells (Ref. 5). The co-occurrence of imbalanced microbial ecosystems with the initiation and advancement of malignant conditions is designated as ‘oncobiosis’. The altered microbiome associated with oncobiosis is termed the ‘oncobiome’ (Ref. 6). This phenomenon of oncobiosis manifests in various neoplastic diseases, including CRC, and potentially exerts a pathogenic influence on the progression of these malignant conditions.

Notably, extensive multicentre studies leveraging advanced technologies are progressively acknowledging the significant role of specific intestinal bacteria in the progression of CRC (Refs 7, 8, 9). For instance, the presence of particular anaerobic bacteria in the gut, like *Fusobacterium nucleatum*, enterotoxigenic *Bacteroides fragilis* (ETBF) and *Bacteroides fragilis*, has been proposed as a potential factor in the genesis of CRC. Their overabundance in the gut environment has been associated with the promotion of inflammation, DNA damage, and the facilitation of tumour growth (Refs 10, 11, 12). Moreover, studies have shown that microbiota-based signatures of the microbiome can be used as promising biomarkers to predict the response to immunotherapy and enhance its effectiveness in treating CRC through modulation (Ref. 13).

The research landscape concerning the association between gut microbiota and CRC metastasis remains limited, with most studies predominantly focusing on examining the correlation between microbial community composition and CRC progression. In this review, we discuss this question, could we consider the gut microbiome as a heavy shotgun for progressing metastasis in CRC?

## The gut microbiota implication in colorectal carcinogenesis

The gut microbiome, often regarded as the ‘forgotten organ’, houses an impressive abundance of over 10^14^ microorganisms, surpassing human cell numbers by tenfold and genomic content by over a hundredfold (Ref. 14). While the gut microbiome performs crucial functions, including maintaining intestinal homeostasis, creating the mucosal immune system, and participating in metabolic processes, extensive multi-omics studies have increasingly linked gut dysbiosis to the development and progression of CRC (Ref. 15). A thorough analysis of microbial genes, strains and functions in stool samples obtained from patients with advanced adenomatous polyps and CRC, as well as healthy individuals, has unveiled significant taxonomical and functional distinctions among the groups. Studies have revealed that patients with CRC display reduced bacterial diversity and abundance in their intestinal mucosa and faeces compared to healthy individuals (Ref. 16). Furthermore, CRC patients exhibit significant alterations in specific bacterial groups such as *B. fragilis*, *F. nucleatum*, *Peptostreptococcus anaerobius*, *Streptococcus gallolyticus*, *Campylobacter* spp., Enterobacteriales spp., and Enterococci, while experiencing a decrease in anti-inflammatory commensal bacteria belonging to the genera *Faecalibacterium*, *Bifidobacterium*, *Lactobacillus*, *Blautia*, *Roseburia* and *Clostridium*. Additionally, it has been demonstrated that patients with early-stage colorectal tumours, progressed adenoma, differ in gut microbiota composition from those with advanced tumours, suggesting a potential role of intestinal microbiota in tumour progression (Ref. 17).

Functionally, changes in the balance of microbial communities can lead to increased permeability of the GI lining, movement of bacteria to other parts of the body, and activation of immune system components that sustain long-term inflammation. The continuous activation of the immune system involves an excessive release of pro-inflammatory cytokines by immune cells, which then trigger signalling pathways within cells lining the colon, mediated by transcription factors such as nuclear factor kappa B (NF-*κ*B) and signal transducer and activator of transcription 3 (STAT3). This also promotes the production of reactive oxygen species (ROS), leading to oxidative stress, damage to DNA and abnormal cell growth. These processes together contribute to the development of colorectal adenomas and cancer (Ref. 18). Both in *vitro* and *in vivo* studies have provided compelling evidence to support the pivotal role of gut dysbiosis/oncobiosis in compromising the integrity of the intestinal barrier and amplifying chronic inflammation, particularly by overgrowth of specific bacteria known as ‘pathobionts’. These pathobionts, in turn, contribute to the initiation of persistent local inflammation, ultimately leading to oncogenic reprogramming and the exacerbation of cancer-like transcriptional phenotypes in the gut, thereby potentiating colonic tumorigenesis. This influence is closely associated with factors such as defects in colonic barrier function, bacterial invasion and the upregulation of inflammatory mediators, including IL-17, Cxcl2, TNF-*α* and IL-1 (Refs 19, 20, 21). For instance, *F. nucleatum*, a widely recognized intestinal pathobiont linked to CRC, induces a pro-inflammatory milieu by upregulating IL-17, leading to the progression of neoplasia in intestinal epithelial cells and the recruitment of immune cells infiltrating the tumour (Ref. 22). *S. bovis* has been found to specifically implicate in the development of CRC by triggering an inflammation-driven sequence of tumour formation or progression. This process involves the interplay of IL-1, IL-8 and cyclooxygenase-2 (COX-2) (Ref. 23). An additional illustration involves ETBF strains that can elicit an elevation in STAT3 response, paired with IL-17-dependent activation of NF-*κ*B. This activation leads to the creation of a mucosal gradient of CXC chemokines, spanning from the proximal to distal regions. Consequently, this gradient facilitates the recruitment of polymorphonuclear immature myeloid cells expressing CXCR2, ultimately resulting in ETBF-induced tumorigenesis in the distal colon (Ref. 24). Colibactin, a genotoxin produced by the polyketide synthetase (pks) genomic island, particularly in B2 phylogroup *E. coli* strains, has been found to cause chromosomal instability and DNA damage in intestinal epithelial cells. This can result in immune cell apoptosis and ultimately contribute to the development of CRC (Ref. 25).

In contrast, the depletion of specific intestinal commensal bacteria with anti-cancer properties and tumour-suppressing metabolites diminishes protective effects against CRC. For example, studies have shown that administering *Faecalibacterium prausnitzii* to azoxymethane-induced CRC rats significantly reduced the occurrence and development of colonic aberrant crypt foci, also lowering lipid peroxidation levels in colon tissues (Ref. 26). Similarly, a combination of *Bifidobacterium longum*, *Bifidobacterium infantis*, *Bifidobacterium breve*, *Lactobacillus plantarum*, *Lactobacillus casei*, *Lactobacillus acidophilus*, *Lactobacillus delbrueckii* subsp. *bulgaricus* and *Streptococcus salivarius* effectively reduced tumour burden in a mouse model induced with azoxymethane/dextran sulphate sodium, accompanied by decreased levels of TNF-*α* and IL-6 in colon tissue (Ref. 27). Additionally, short-chain fatty acids (SCFAs) like propionate and butyrate, synthesized by intestinal lactobacilli and bifidobacteria, have been demonstrated to enhance apoptosis, regulate immune responses within colorectal tissues and inhibit carcinogenesis and tumour growth (Ref. 28). Moreover, Gpr109a, a receptor present in the colon responding to butyrate and niacin, promotes anti-inflammatory properties in colonic macrophages and dendritic cells by inducing the differentiation of regulatory T cells (Treg cells) and IL-10-producing T cells (Ref. 29).

The gut microbiome has long been associated with the development of CRC. Dysbiosis in the gut, marked by changes in microbial composition, can prompt inflammation and play a role in the progression of CRC. Certain bacteria, referred to as ‘pathobionts’, have been linked to chronic inflammation and the advancement of CRC, while beneficial commensal bacteria and their by-products have demonstrated anti-tumour properties. These findings underscore the intricate interplay between gut microbiota, inflammation and CRC, offering valuable insights into the progression of this disease.

## Emerging insights into gut microbiome's role in cancer metastasis

The occurrence of oncobiosis in the gut has emerged as a crucial factor driving the metastasis and dissemination of tumour cells in recent studies. Primarily, the disproportionate representation of specific oncogenic bacteria, including *F. nucleatum* (Ref. 30) and *Porphyromonas gingivalis* (Ref. 31), as well as the reduction of beneficial commensals like *Bifidobacteriaceae* (Ref. 32) and *Akkermansia muciniphila* (Ref. 33), coupled with changes in the production of microbial metabolites like cadaveric amines, indolepropionic acid (IPA) and sodium butyrate, have been described to contribute to the metastasis of various malignancies through multiple mechanisms (Refs 34, 35).

The transformation in cell phenotype that promotes the acquisition of migratory and invasive characteristics in cancer cells, ultimately resulting in the formation of metastatic foci, can be intensified through various mechanisms, encompassing epigenetic modifications such as epithelial–mesenchymal transition (EMT), genetic abnormalities like mutations in the DNA damage response (DDR), microenvironmental factors such as immune cell activation-induced by cancer, and physical factors such as fluid shear stress (FSS) (Ref. 36). Gut microbiota can trigger EMT and enhance the spread of tumour cells by reducing *β*-linked proteins and initiating the Wnt/*β*-catenin signalling pathway (Ref. 37). Certain bacterial components, including lipopolysaccharide (LPS), flagellin and adhesins, possess the ability to bind to the junctional proteins E-cadherin and ZO-1 found in epithelial cells. This specific interaction leads to a disturbance in cellular polarity and subsequently alters the downstream Wnt/*β*-catenin signalling pathway, ultimately culminating in EMT (Ref. 38). Through an alternative pathway, the presence of *P. gingivalis*, *F. nucleatum* and *Streptococcus gordonii* within the oral cavity is able to prompt the promoter activity and nuclear localization of the ZEB1 transcription factor. This, in turn, leads to an elevation in the expression of different mesenchymal markers, including vimentin and MMP-9 (Ref. 39). Moreover, there is evidence suggesting that certain microbes, including ETBF, stimulate the generation of ROS within intestinal epithelial cells. This heightened ROS production, in turn, triggers the activation of matrix metalloproteinases (MMPs), ultimately inducing EMT programmes in epithelial cells. Consequently, leads to an enhancement of tumour cell invasion and metastasis (Ref. 40). Conversely, a balanced microbiome composition can boost the expression of junctional proteins E-cadherin and ZO-1 through various mechanisms, preventing EMT induction and promoting autophagy (Ref. 41).

Additionally, the association of gut microbial composition with populations of immune cells has been also investigated, revealing a significant association between microbial diversity and the immune response (Ref. 42). Immune cells such as M2-polarized macrophages and T helper 17 (Th17) cells have been implicated in cancer metastasis (Ref. 43). During the metastatic process, the gut microbiota plays a significant role in aiding tumour cells in adjusting to the biochemical and physical factors within the tumour microenvironment. One notable physical factor that contributes to tumour cell metastasis is shear stress caused by FSS (Ref. 44). Scientific research has demonstrated that the microbiome can influence the actin cytoskeleton, ultimately making tumour cells more resistant to FSS (Ref. 45). Furthermore, some microbial species have a selective impact on MMPs, including MMP-1/9/10 and MMP-2F/9F, with the former promoting cancer metastasis (Ref. 31) and the latter suppressing it (Ref. 46).

DDR system plays a critical role in safeguarding the integrity of genetic material against various forms of damage and supporting the survival of cancer cells. Under specific circumstances, dysbiotic microbes can interfere with the activation of DDR in host cells, resulting in genetic mutations or epigenetic alterations in crucial genes that are closely linked to the processes of tumour invasion and metastasis (Ref. 47). DDR activation can be initiated through two mechanisms: either directly by the genotoxic effects of microbe-produced substances, primarily the cytolethal distending toxins (CDT) released by certain Gram-negative bacteria in the intestinal tract, or indirectly through the generation of ROS or reactive nitrogen species (RNS) resulting from prolonged or excessive activation of the host immune cells in response to bacteria or their metabolic by-products (Ref. 47).

The complex interaction between the gut microbiome and tumour metastasis is a rapidly advancing field with profound implications for cancer research and treatment. Understanding the roles of specific oncogenic bacteria, beneficial commensals, microbial metabolites and immune responses in promoting metastasis offers potential avenues for therapeutic interventions. Moreover, the influence of dysbiotic microbes on genetic and epigenetic processes linked to tumour invasion underscores the need for further exploration of microbiome-targeted strategies to mitigate cancer metastasis. This growing body of knowledge highlights the potential for novel microbiome-based approaches in the fight against cancer metastasis.

## The Gut microbiota's critical role in facilitating CRC invasion and metastasis

Emerging evidence suggests that the gut microbiota could play a potential role in the invasion and metastasis of CRC. *F. nucleatum* has been previously recognized to be significantly enriched in CRC patients with lymph node metastasis compared to those without lymph node metastasis (Ref. 48). Accordingly, *F. nucleatum* infection enhanced the migration of CRC HCT-116 and LoVo cell lines and increased lung metastasis in nude mice. Remarkably, the presence of *F. nucleatum* has been validated in the metastatic lung lesions of nude mice using FISH assay (Ref. 48). The presence of *F. nucleatum* is also correlated with heightened self-renewal and proliferation of colorectal cancer stem-like cells (CCSCs), leading to the onset of CRC, metastasis and resistance to chemotherapy (Ref. 49). Additionally, *F. nucleatum* exhibited control over diverse lipid metabolisms in both CCSCs and CRC cells (Ref. 49). In CCSCs, *F. nucleatum* facilitated fatty acid oxidation (FAO), resulting in a decrease in lipid accumulation. This, in turn, provided the necessary energy for the self-renewal and proliferation of CCSCs. Conversely, in regular CRC cells, *F. nucleatum* stimulated the synthesis of fatty acids and triacylglycerols, leading to an increase in lipid droplet levels. These accumulated lipid droplets induced the activation of the Notch signalling pathway by enlisting the Notch inhibitor Numb for degradation. Accordingly, it can be definitively inferred that *F. nucleatum* advances the progression of metastatic CRC (mCRC) by manipulating its stemness characteristics. Chen *et al*. recently conducted a study to explore the impact of gut microbiota on treatment outcomes for patients with mCRC (Ref. 50). The study included 110 mCRC patients who showed progressive disease (PD) or partial response (PR) for at least 12 cycles of therapy. These patients were further divided into subgroups based on the type of targeted therapy received, either anti-epidermal growth factor receptor (cetuximab) or anti-vascular endothelial growth factor (bevacizumab). They found that the PR group and bevacizumab-PR subgroup had significantly higher *α*-diversity in their gut microbiota. There was also a significant difference in the *β*-diversity of bacterial species between the bevacizumab-PR and bevacizumab-PD groups. Among the bacterial species analysed, *Klebsiella quasipneumoniae* showed the largest difference in abundance between the PD and PR groups. Lactobacilli and Bifidobacteria were more abundant in the PD group, while the abundance of *F. nucleatum* was approximately 32 times higher in the PD group compared to the PR group. The study concluded that a higher diversity of gut microbiota was associated with more favourable treatment outcomes in patients with mCRC. However, the analysis of bacterial species in the stool samples yielded heterogeneous results.

Recent research has presented evidence that various groups of cancer cells originating from the primary tumour possess the capability to migrate to distant organs. This suggests that the dissemination of distant metastases may occur through the bloodstream independently of lymph node involvement (Refs 51, 52). The portal vein serves as a direct conduit for venous blood from the intestines to reach the liver, making it the most probable route for CRC tumour cells to spread via the bloodstream (Ref. 53). Bertocchi and colleagues have uncovered that a higher presence of plasmalemmal vesicle associated protein-1 expressing cells (PV-1 + ) in the primary tumour of CRC patients indicates increased blood vessel permeability, which is linked to the occurrence of distant metastases over time (Ref. 54). Their findings underscore PV-1 as a potential prognostic indicator for distant metastases. Additionally, combining the evaluation of PV-1 status and lymph node involvement in CRC patients offers a more precise prediction of distant recurrence compared to assessing these factors independently.

**Figure 1.**
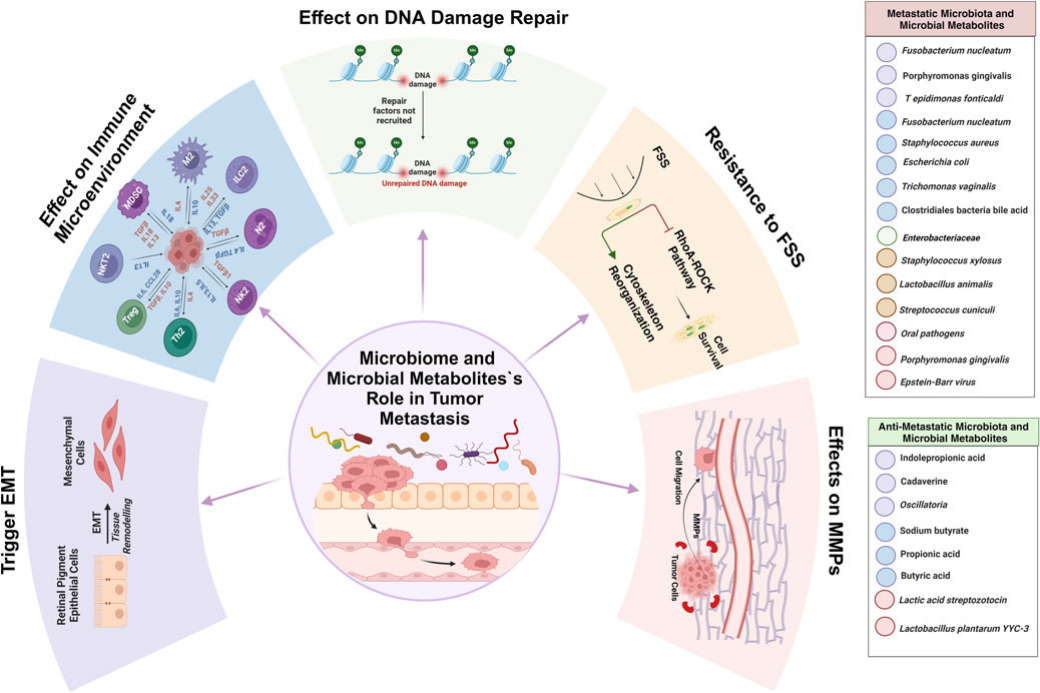
The impact of some key gut microbiota and their metabolites on tumour invasion and metastasis across diverse pathways. The gut microbiota composition, particularly the presence of oncogenic bacteria like *F. nucleatum* and *P. gingivalis*, alongside diminished beneficial commensals such as *Bifidobacterium* and *Lactobacillus* spp., coupled with changes in microbial metabolites, can contribute to tumour metastasis through various mechanisms. Pre-existing GI dysbiosis, often triggered by antibiotic use, has been shown to facilitate tumour growth and spread in animal models. Alterations induced by gut microbiota can promote epithelial–mesenchymal transition (EMT), affecting the spread and invasiveness of cancer cells. The microbiome's interaction with immune cells, as well as its influence on the expression of MMPs, FSS resistance and DNA damage response activation, play crucial roles in cancer progression and metastasis. These findings underscore the intricate relationship between gut microbiota dysbiosis and cancer metastasis, highlighting potential therapeutic targets for intervention.

In their investigation, the researchers delineated a series of steps leading to the progression of liver metastases. This sequence commences with bacteria infiltrating the tumour tissue and modifying the gut vascular barrier (GVB). Subsequently, these bacteria migrate to the liver and facilitate the formation of polymorphonuclear (PMN) leucocytes, which create a conducive environment for the spread of cancer cells. Progress has been made in understanding the specific bacteria and molecular mechanisms involved in this process. The research findings have disclosed that a specific strain of *E. coli* C17 has the capacity to directly compromise the GVB by utilizing a type III secretion system (TTSS) virulence factor (Virf), enabling it to translocate into the liver. Once in the liver, this strain of *E. coli* stimulates the recruitment of immune cells, supporting the maturation of the PMN and promoting the formation of metastases. Significantly, evidence has been revealed of the same strain of *E. coli* C17 in both the primary tumour and liver metastases of individuals with human CRC. These findings suggest that a similar mechanism may be in operation in human CRC and validate a previous report indicating that identical bacteria colonize both primary colon cancer and paired liver metastases. Figure 1 provides a schematic representation illustrating the influence of gut microbiota and their by-products on tumour invasion and metastasis.

### Influential role of gut microbial metabolites in driving CRC metastasis

The gut microorganisms generate metabolites when they absorb specific substances. The metabolites produced by gut microbiota comprise various compounds such as small volatile molecules, lipids, proteins, peptides, sugars, secondary bile products or terpenes, biogenic amines, oligosaccharides, glycolipids, organic acids and amino acids. These metabolites serve as both signalling molecules and components for metabolic reactions. Accumulating evidence suggests that microbial metabolites could also be a key event in the development and pathogenesis of CRC, not solely limited to the pro-carcinogenic activities of an individual pathogen but also during the impact of a broader microbial community, called ‘gut metabolome’ (Refs 55, 56). Newly available reports indicate that SCFAs, including acetate, propionate and butyrate, contribute to reducing inflammation and inhibiting cancer development (Ref. 57). On the other hand, certain microbial metabolites like secondary bile acids have been found to promote the growth of cancer cells (Ref. 58). Formerly, a research study has demonstrated that the increased presence of natural killer T cells, in response to the production of secondary bile acids by Clostridiales bacteria, can provide protection against hepatocellular carcinoma (HCC) and liver metastases, which are commonly derived from mCRC (Ref. 59). Butyrate, another important metabolite produced by colonic anaerobic bacteria (such as *F. prausnitzii*, *Roseburia intestinalis* and *Butyrivibriocrossotus*), has multiple functions in the aetiology of CRC. It acts as an inhibitor of histone deacetylase (HDAC), leading to increased histone acetylation, and plays a role in CRC cell apoptosis and proliferation (Ref. 60). Furthermore, butyrate hinders angiogenesis, metastasis and cell survival in CRC by inhibiting the neuropilin-1/vascular endothelial growth factor pathway through the suppression of Sp1 transactivation (Ref. 61). It also reduces cell proliferation, clone formation and migration by promoting the secretion of endocan via the ERK2/MAPK pathway (Ref. 62). In addition, butyrate induces apoptosis in CRC cells by inhibiting the Wnt/*β*-catenin signalling pathway and enhances the effectiveness of anticancer therapies by modulating the immune response of cytotoxic CD8 + T cells (Ref. 63). Hence, butyrate, as a crucial metabolite of the gut microbiome, could serve as a protective agent against the progression of mCRC.

Lithocholic acid (LCA), predominantly produced by Clostridiales, is a secondary bile acid that acts as an endogenous promoter of CRC (Ref. 64). LCA triggers the production of ROS in the GI tract, causing damage to the protective epithelial layer by reducing cell death, increasing cell multiplication, inducing oxidative DNA damage, provoking inflammatory responses and activating the NF-*κ*B signalling pathway (Ref. 65). Moreover, LCA influences the muscarinic 3 receptor and Wnt/*β*-catenin signalling in colonic epithelial cells, promoting the development of cancer stem cells associated with CRC (Ref. 66). LCA also enhances the expression of IL-8, which activates the ERK1/2/mitogen-activated protein kinase (MAPK) signalling pathway, suppresses the activity of STAT3, and stimulates angiogenesis associated with CRC (Ref. 67). Furthermore, LCA treatment results in the upregulation of genes involved in tumour invasion and metastasis, such as MMPs – MMP-1, MMP-2 and MMP-7, and stimulation of urokinase plasminogen activator receptor, which contributes to the invasive and malignant properties of cancerous epithelial cells (Ref. 68).

The gut bacteriome plays a significant role in the metabolism of amino acids, particularly serine and tryptophan, which have a profound impact on the survival of tumour cells, cell migration and progression of mCRC. As an illustration, it has been observed that phosphoglycerate dehydrogenase (PHGDH) undergoes monoubiquitination by a cullin 4A-based E3 ligase complex at lysine 146 in CRC cells. This monoubiquitination leads to an increase in PHGDH activity by enlisting the assistance of a chaperone protein, DnaJ homologue subfamily A member 1. The chaperone protein promotes the formation of PHGDH tetramers, resulting in higher levels of serine, glycine and *S*-adenosylmethionine (SAM). The elevated SAM levels, in turn, upregulate the expression of cell adhesion genes (laminin subunit gamma 2 and cysteine-rich angiogenic inducer 61) by initiating histone H3K4 trimethylation mediated by SET domain containing 1A. Consequently, this promotes the migration of tumour cells and metastasis in CRC. Interestingly, the levels of SAM in tumours or blood samples have shown an association with the recurrence of metastasis in CRC patients (Ref. 69).

It is worth mentioning that the connection between gut microbiota and their metabolite and mCRC is intricate and still not completely comprehended. More investigation is required to completely clarify the involvement of gut microbiota in the advancement and management of mCRC.

### Microbiome composition and impact within the tumour microenvironment of CRC

Tumour microenvironment (TME) refers to a multifaceted and dynamic entity comprised of organs, tissues, their functions and the metabolic processes within. The TME has a strong correlation with tumour progression, development and metastasis (Ref. 70). The role of the microbiome in promoting tumorigenesis has gained attention due to the presence of microorganisms within tumour tissues. However, the analysis of the tumour-associated microbiome poses a significant challenge due to microbial contamination. Several studies have characterized distinct compositions of the microbiome in the proximal and distal regions of a tumour, indicating the existence of a tumour-specific microbiome within the tumour microenvironment (Ref. 71). CRC can be characterized as a malignancy infiltrated by effector memory lymphocytes, and the TME plays a pivotal role in cancer immunotherapy. Within the TME of CRC, various components exist, including tumour cells, blood vessels, extracellular matrices, fibroblasts, lymphocytes, bone marrow-derived suppressor cells and signalling molecules (Ref. 72). Certain microorganisms proliferate within TME and exert their influence on tumour progression. Consequently, specific microorganisms have been found to have a positive impact on the TME, thereby eliciting anti-cancer effects. At present, immunotherapies for CRC, such as programmed cell death protein 1 (PD-1) or cytotoxic T lymphocyte-associated antigen-4 (CTLA-4) inhibitors, primarily function through T cells. Conversely, beneficial bacteria synergistically collaborate with immune checkpoint blockade (ICB) by activating immune cells and regulating cytokine secretion (Ref. 73). *F. nucleatum* induces the expression of tumour-derived C-C motif chemokine ligand 20 (CCL20), facilitates the attraction of macrophages and immunosuppressive myeloid-derived suppressor cells (MDSC) within the TME, and converts macrophages into the M2 subtype. These actions lead to the development of an immunosuppressive microenvironment that promotes tumour progression and facilitates the metastasis of CRC (Ref. 74).

### Gut microbiota's influence on epigenetic alterations in CRC metastasis

The metastatic process of mCRC entails a complex interplay of molecular and phenotypic alterations that are essential for the successful dissemination of tumour cells. While substantial advancements have been made in comprehending the genetic factors involved in the initiation of cancer, our understanding of the genetic events propelling cancer metastasis remains somewhat limited (Ref. 75). More recently, there has been a growing realization among researchers regarding the significance of non-mutational epigenetic reprogramming as a fundamental mechanism that endows tumour cells with the necessary characteristics for metastatic progression. Epigenetic mechanisms, including DNA methylation, histone modifications and regulation by non-coding RNAs, are now recognized as pivotal drivers of the metastatic cascade (Refs 76, 77, 78). Considering the dynamic nature of epigenetic changes, it is highly plausible that these events occupy a central role in CRC metastasis. Notably, recent investigations have furnished compelling evidence establishing a connection between epigenetic alterations, CRC metastasis and reduced patient survival (Refs 79, 80, 81). The realm of epigenetics has illuminated our understanding of how environmental factors can intricately influence the intricate mechanisms governing gene expression. Intriguingly, emerging research posits that metabolites synthesized by the gut microbiota may exert a substantial influence on epigenetic alterations. This revelation heralds novel opportunities to investigate the intricate interplay between the gut microbiota and the genesis of mCRC. Keeping these perspectives in mind, our present endeavour is to scrutinize the modulatory impact of the gut microbiota and its metabolites on epigenetic modifications within the intricate framework of mCRC. A thorough summary of relevant research delving into the involvement of gut microbiota in epigenomic alterations associated with mCRC is thoughtfully presented below. It highlights the diverse gut microbiota and their respective roles in driving epigenomic changes in CRC, encompassing the type of modification, underlying mechanisms, clinical implications, metastasis procedures and key references. Stay informed as ongoing research in microbiota-associated epigenomics in CRC unfolds, offering valuable insights into comprehending and potentially manipulating the disease. Table 1 provides a summary of the key gut microbiota and their consequential effects on epigenetic alterations which have the potential to influence the aggression and spread of CRC.

**Table 1.** A summary of the key gut microbiota and their consequential effects on epigenetic alterations which have the potential to influence the development and dissemination of CRC

Microbe/Microbial factor(s)	Epigenetic modifications	Metastatic or antimetastatic effect	Interaction with DNA methylation	Interaction with histone modification	Study type	References
*Fusobacterium*	DNA methylation; Microsatellite instability.	Metastatic	Suppression of the METTL3 via activation of YAP pathway and inhibition of FOXD3 expression; type C methylation of MINT2 and MINT31; Hypermethylation of MLH1.	–	*In vitro*; *In vivo*; Clinical	(Ref. 92)
*L. monocytogenes*	Histone modifications	Metastatic	–	Deacetylation of histone H3K18 by introducing the SIRT2 into the nucleus of host cells through the interaction of bacterial InlB with c-Met on the host cell surface.	*In vitro*; *In vivo*	(Ref. 150)
*Enterococcus*	DNA methylation	Metastatic	Methylation of promoter of tumour suppressor gene SFRP2	–	*In vivo*	(Ref. 151)
Enterobacterales spp.	DNA methylation	Metastatic	DNA methylation induced by pro-inflammatory-related pathways	–	*In vitro*; *In vivo*	(Ref. 91)
*Bifidobacterium* and *Lactobacillus* spp.	DNA methylation	Antimetastatic	Production of methyl donors modifying the methylation pattern of prometastatic genes	–	*In vitro*; *In vivo*; Clinical	(Ref. 58)
Butyrate, acetate, and propionate	DNA methylation; Histone modifications	Antimetastatic	Influences DNA methylation patterns.	Influencing histone modifications through their metabolic conversion into acetyl-CoA	*In vitro*; *In vivo*	(Refs 127, 128, 129)
Choline-derived metabolites	DNA methylation	Antimetastatic	Inhibiting hypermethylation-mediated inactivation of critical growth factors and prometastatic genes	–	*In vitro*, *in vivo*	(Ref. 100)
Folate	DNA methylation	Antimetastatic	Inhibiting hypermethylation-mediated inactivation of critical growth factors and prometastatic genes	Regulates gene expression; impacts CRC progression	*In vitro*, *in vivo*	(Ref. 103)

#### Interplay of gut microbiota and DNA methylation in CRC metastasis

Aberrant DNA methylation is widely recognized as a characteristic feature of mCRC progression and is implicated in the dysregulation of key driver genes. A comprehensive investigation of the DNA methylation patterns in circulating tumour cells that possess metastatic abilities in CRC has identified a distinct DNA methylation signature that could bear relevance to the formation of metastases (Ref. 82). Notably, the hypermethylation patterns observed in CRC and liver metastases closely resemble those observed in primary CRC, suggesting a potential association between DNA methylation and mCRC (Ref. 83). The fascinating and rapidly evolving realm of epigenetics has brought to light the captivating influence of gut microbiota on DNA methylation. DNA methylation, a critical epigenetic modification, involves the addition of a methyl group to the DNA molecule, primarily targeting cytosine residues within CpG dinucleotides (Refs 84, 85). This essential process exerts profound control over gene expression and is implicated in a multitude of biological phenomena, spanning developmental processes, cellular differentiation and disease aetiology. Recent investigations have delved into the intricate interplay between the resident gut microbiota and the host epigenome, specifically unraveling the impact of these interactions on DNA methylation patterns (Refs 86, 87, 88, 89). Noteworthy observations shed light on the distinctive gut microbiota signature and its consequential effects on DNA methylation dynamics. The GI milieu serves as a pivotal conduit for both nutrient assimilation and metabolic pathways, while concurrently exerting a profound influence on the multifaceted epigenetic framework. Within this context, microbial-derived metabolites, particularly SCFAs generated through the gut bacterial fermentation of dietary fibres, have been identified as integral modulators of DNA methylation profiles (Ref. 87). The intricate interrelationship linking the gut environment and epigenetic modifications has emerged as a prominent subject of scientific scrutiny. Extending beyond its conventional role in nutrient absorption and metabolism, the GI tract has garnered recognition as a dynamic orchestrator of the epigenetic panorama. Among the key actors in this intricate symbiosis is the gut microbiota, a diverse assemblage of microorganisms inhabiting the GI domain.

Alterations occurring within the community-wide and individual-specific colonic microbiota have been recently correlated with host epigenomic alterations. In a study conducted by Tahara *et al*., ulcerative colitis (UC) patients with high levels of *Fusobacterium* enrichment exhibited a pronounced degree of type C (cancer-specific) methylation compared to other samples with lower or negligible levels of *Fusobacterium* (Ref. 90). Potentially, genes implicated in mCRC and acknowledged for type C methylation, including MINT2, MINT31, P16 and NEUROG1, displayed heightened methylation levels upon exposure to a high frequency of *Fusobacterium* (Ref. 90). Through multivariate analysis, it was determined that the *Fusobacterium* enriched status independently increased the likelihood of high methylation. More recently, Ryan *et al*. described that modifications in epithelial DNA methylation patterns not only enhance disease classification but also demonstrate correlations with both inflammation and the composition of the microbiota in the colon (Ref. 91). Within the microbiota sub-groups identified, a dominant presence of Enterobacterales and *Bacteroides* species has been observed, both of which are representative of strains known to exhibit pro-inflammatory characteristics in vitro studies. Furthermore, these microbiota sub-groups are also associated with immune-related epigenetic markers. In conclusion, their study demonstrates discernible disparities in both microbiota composition and epigenetic profiles between inflamed and non-inflamed segments of the colon, regardless of whether the subjects were diagnosed with IBD.

In a recent investigation led by Chen and colleagues, it was uncovered that *F. nucleatum* exerts a pivotal influence on the diminishment of N6-methyladenosine (m6A) modifications in CRC cells, consequently contributing to the promotion of CRC aggressiveness (Ref. 92). This reduction is ascribed to the suppression of the methyltransferase-like 3 (METTL3), the major m6A methyltransferase, by *F. nucleatum*, consequently triggering the initiation of CRC metastasis. The investigators pinpointed the forkhead box D3 (FOXD3) transcription factor as the key regulator of METTL3. *F. nucleatum* activates the YAP signaling pathway, which, in turn, suppresses FOXD3 expression, leading to diminished METTL3 transcription. The decreased levels of METTL3 result in elevated expression of its downstream target gene, kinesin family member 26B (KIF26B), by altering its m6A modifications and compromising YTHDF2-mediated mRNA degradation. These alterations contribute significantly to the facilitation of CRC metastasis induced by *F. nucleatum*. Additionally, the expression of METTL3 is negatively correlated with *F. nucleatum* and KIF26B levels in CRC tissues (Ref. 92). Moreover, high levels of KIF26B are significantly associated with shorter survival times in mCRC patients (Ref. 92). These findings shed light on the modulation of the human m6A epitranscriptome by gut microbiota and its significance in mCRC.

DNA methylation patterns emerged as a crucial regulatory element within this intricate framework, underscoring the wider implications of gut microbiota-derived metabolites in maintaining immune system homeostasis. Recent scientific investigations have provided valuable insights into the profound impact of gut microbial metabolites, particularly SCFAs, on the intricate patterns of DNA methylation (Ref. 86). The complex bidirectional communication between the gut microbiota and the host epigenome was meticulously examined in the context of both physiological well-being and pathological conditions (Ref. 93). By synthesizing a wealth of compelling evidence, it could consolidate noteworthy findings, demonstrating that gut dysbiosis can induce alterations in DNA methylation profiles associated with various pathological manifestations, including IBD and metabolic disorders. The influence of SCFAs on DNA methylation has garnered increasing attention in the field of epigenetic research. Recent studies have provided valuable insights into the intricate association between SCFAs, especially butyrate, and DNA methylation, shedding light on their potential effects on gene expression (Ref. 94). The site-specific effects of butyrate on DNA methylation within the context of cancer aggressiveness has been described previously. It has been demonstrated that treatment with butyrate caused alterations in DNA methylation patterns at specific genomic loci, subsequently influencing the expression of genes associated with cellular proliferation and apoptosis (Ref. 94). This study further underscored the potential therapeutic implications of targeting DNA methylation through the modulation of SCFAs in the context of cancer treatment. Furthermore, the impact of propionate on DNA methylation patterns within the context of metabolic diseases, particularly diabetes, revealed that gut microbiota-derived propionate induces specific DNA methylation, thus predisposing obesity-prone individuals to diabetes (Ref. 95). These findings suggest a potential link between gut microbiota-derived SCFAs, epigenetic modifications and the maintenance of metabolic homeostasis. Consequently, it is pertinent to emphasize the need for further investigations into the potential therapeutic applications of SCFAs in cancer treatment, particularly in the context of mCRC.

#### Impact of gut microbiota on methyl donor metabolism and epigenetic signatures in CRC metastasis

Scientific investigations have revealed that the insufficiency of methyl donors can exert substantial effects on vital metabolic pathways and perturb the composition of metabolites within the colon. These alterations possess the potential to impede the progression of CRC as well as mCRC (Ref. 96). Epigenome-wide investigations across various malignancies have demonstrated that methyl group donors exhibit antimetastatic effects by inducing hypermethylation-mediated inactivation of critical growth factors and prometastatic genes (Refs 97, 98). The impact of gut microorganisms on the metabolism of dietary components, specifically methyl donors such as folate, choline and methionine, has become a central focus in comprehending the complex correlation between gut microbiome and DNA methylation. Recent studies documented the dynamic interaction between the composition of the gut microbial community, dietary methyl donors and epigenetic mechanisms (Ref. 99). Notably, the microbial breakdown of choline, a significant methyl donor has been found to have essential roles in biology as a methyl donor (Ref. 100). Recent research has provided considerable evidence regarding the selective participation of specific strains of gut microorganisms in the metabolic processes of choline. As a consequence, these metabolic activities give rise to the production of metabolites which exert a subsequent influence on the physiological functions of the host organism (Ref. 101). These choline-derived microbial metabolites were observed to exert influence on DNA methylation patterns in the host, establishing a connection between metabolic activities of the gut microbiota and epigenetic modifications (Ref. 102). The association between the composition of gut microorganisms and folate metabolism, an essential provider of methyl groups in one-carbon metabolism, has been recently elucidated (Ref. 103). This evidence suggests that specific taxa of gut microorganisms actively partake in folate metabolism, subsequently impacting the availability of this crucial methyl donor. Moreover, these studies emphasize how changes in the composition of the gut microbiota can result in alterations in folate metabolism, thereby influencing the intricate process of DNA methylation. This suggests that the molecular alterations orchestrated by oncobiosis are believed to extend beyond solely influencing DNA methylation directly. They could potentially have significant impacts on the pool of methyl donors and methylating agents, which could in turn account for a substantial portion of the gene expression modifications related to metastasis and tumour aggressiveness. Importantly, future in-depth studies should be undertaken to elucidate the precise mechanisms underlying the epigenomic alterations triggered by methyl donors linked to gut microbiota in mCRC.

#### The influence of gut microbiota on CpG island methylator phenotype (CIMP) in CRC metastasis

CRC can be classified into a distinct subgroup referred to as the CpG island methylator phenotype (CIMP), which emerges from the disruption of epigenetic stability. This subtype is characterized by widespread hypermethylation at CpG island sites within gene promoters, resulting in the inactivation of numerous tumour suppressor genes and other genes associated with tumorigenesis (Ref. 104). This hypermethylation may also play a role in the progression and advancement of mCRC (Refs 105, 106). Identification of CIMP-positive tumours typically involves assessing a panel of up to 16 genes, including commonly used methylation markers such as CACNA1G, IGF2, NEUROG1, RUNX3, SOCS1, CRABP1, MINT1, MINT2 and MINT31 (Ref. 107). The CIMP pathway is closely associated with microsatellite instability (MSI) due to hypermethylation of MLH1, with a higher frequency observed in serrated tumours and the proximal colon of older patients (Ref. 108). The impact of the intestinal microbiota on CIMP in various cancers has become a compelling area of research. A significant focus of extensive research has been to explore the potential influence of the microbiota on DNA methylation, particularly in CIMP-positive tumours and the bacterium *F. nucleatum* (Refs 109, 110, 111). In a research study conducted by Tahara and colleagues, elevated levels of Fusobacterium were found to be linked with CIMP positivity in CRC tissues, as evidenced through the analysis of seven markers: ER, SFRP1, MYOD1, MGMT, SLC16A2, SPOCK2 and N33 (Ref. 112). Similar findings, indicating a strong correlation between various markers and CIMP, have been reported by other authors as well (Refs 111, 113, 114). However, Park *et al*. did not find a significant association when they analysed eight markers (MLH1, NEUROG1, CRABP1, CACNA1G, CDKN2A, IGF2, SOCS1 and RUNX3) (Ref. 113) or CIMP diagnosis (Ref. 115). They did find a significant association between high levels of *F. nucleatum* and CDKN2A methylation in CRC tissues with high MSI. Furthermore, some studies have reported an association between MLH1 hypermethylation and an abundance of *F. nucleatu*m (Ref. 110). Additionally, Hamada *et al*. observed a similar distribution of LINE-1 methylation levels and *F. nucleatum* in both negative and positive tumours for MSI-high (Ref. 116). Other species such as *B. fragilis*, *Faecalibacterium prausnitzii* and *E. coli* have also been examined, but no relationship has been described with CIMP (Ref. 113).

#### Microbial impact on histone modifications and epigenetic regulation in CRC metastasis

In various human malignancies, shifts in histone modification patterns, specifically the acetylation of distinct lysine residues on histones, have been observed to be associated with tumour aggressiveness and metastatic progression, alongside changes in DNA methylation (Ref. 117). This acetylation process is tightly regulated by enzymes known as histone acetyltransferases (HATs) and histone deacetylases (HDACs) (Refs 118, 119). HATs facilitate the transfer of acetyl groups from acetyl coenzyme A (Ac-CoA) to lysine residues on histones, whereas HDACs reverse this process. Consequently, HATs and HDACs influence CRC progression and aggressiveness through modulation of the acetylation status of crucial proteins such as p53 and tubulin (Ref. 120).

Microbial organisms and their metabolic products have been recognized for their significant influence on orchestrating alterations in histone modifications within the host's tissues through diverse and intricate pathways. Studies have demonstrated that infection with *Listeria monocytogenes* triggers the elimination of acetyl groups from histone H3K18 in colonic cells, leading to impactful modifications in the expression of various genes, including SMAD1, IRF2, SMARCA2 and CXCL12 (Ref. 121). This bacterium achieves deacetylation by transporting the deacetylase sirtuin 2 (SIRT2) into the host cell nucleus via the interplay of its virulence factor InlB with the binding of c-Met on the host cell surface, effectively regulating the expression of genes associated with the cell cycle and modulating the host's immune response to promote its invasion. (Ref. 122). For instance, SMAD1, a member of the SAMD family, has been associated with the metastatic progression and migratory behaviour of CRC cells by facilitating the concurrent expression of Snail/Ajuba proteins. Notably, activation of SMAD1 results in the downregulation of E-cadherin, a documented downstream target gene of Snail/Ajuba. Additionally, the reduction of Ajuba in HCT116 cells significantly diminishes the migratory potential induced by Smad1 upregulation, emphasizing the critical role of Ajuba in enabling SMAD1-mediated cell migration. Importantly, specific bacterial metabolites, particularly SCFAs, possess the capacity to induce histone modifications in colonic cells (Refs 123, 124). Butyrate, a by-product of dietary carbohydrate and protein fermentation by Firmicutes bacteria, exerts regulatory effects on a significant number of genes by histone modifications, including those involved in apoptosis and cell cycle regulation, with an impact on over 4000 genes (Ref. 125). Firmicutes bacteria have the ability to influence the activity of HDACs through the production of SCFAs (Ref. 126). Groundbreaking investigations by Smith *et al*. have shed light on the inhibitory role of SCFAs, particularly butyrate, on histone deacetylases (Ref. 94). Another study conducted by Fellows *et al*. delved into the molecular mechanisms by which butyrate, a prominent SCFA, exerts its influence on histone crotonylation in the colon (Ref. 127). The research findings revealed that butyrate functions as an inhibitor of histone deacetylases, thereby preventing the removal of acetyl groups from histone proteins. This evidence emphasized the significance of butyrate-induced histone modifications in orchestrating the dynamics of DNA methylation, thereby highlighting the interconnected nature of these epigenetic processes. Furthermore, the meticulous investigation conducted by Li *et al*. has provided valuable insights into the essential role of SCFAs in modulating immune responses through epigenetic mechanisms (Ref. 128). Additionally, SCFAs are capable of influencing histone modifications through their metabolic conversion into acetyl-CoA and other precursors of SCFA-CoA. These SCFA-CoA precursors can then be effectively transferred to histones, mediated by HATs such as p300/CBP (Ref. 129). Using mass spectrometry analysis, Krautkramer *et al*. investigated the effects of the microbiota and diet on histone modifications (Ref. 130). They utilized conventionally raised, germ-free and microbiota-recolonized mice to study the role of the microbiota. The study revealed that the gut microbiota influences histone acetylation and methylation in the colon, liver and white adipose tissue. SCFAs produced by the microbiota play a major role in this process. Histone acetylation increased significantly in various tissues with the presence of the microbiota, while H3 methylation showed more subtle changes. Some histone modifications were consistently regulated across all tissues, while others varied by tissue.

**Figure 2.**
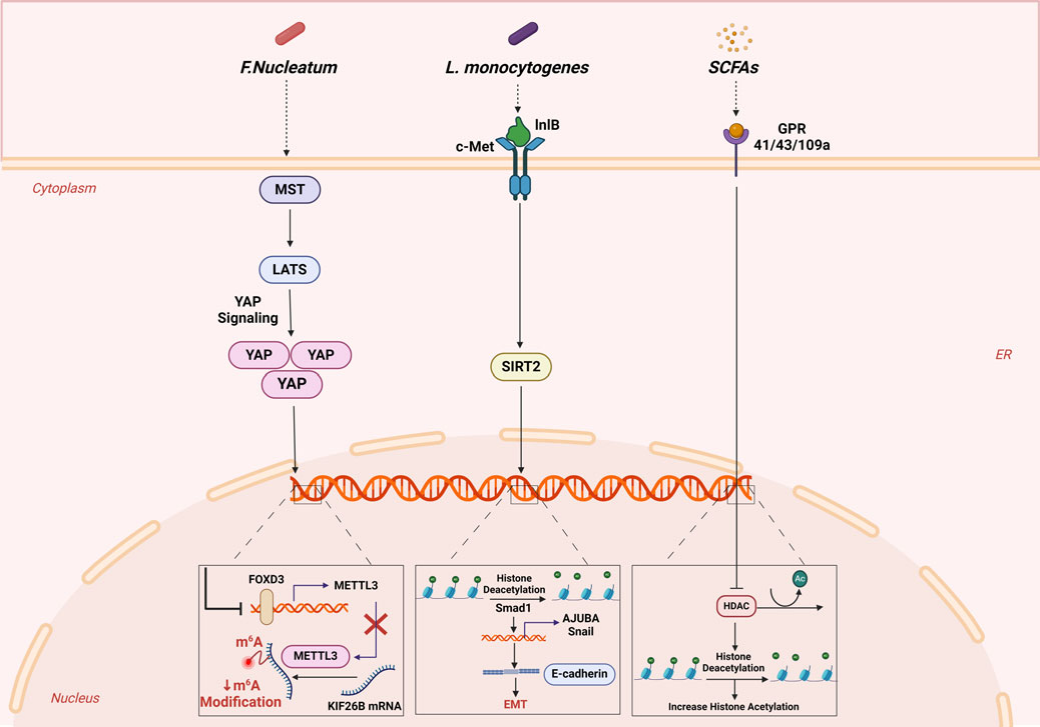
A subset of microbiota and microbial metabolites that modulate pathways associated with epigenetic modifications pertinent to CRC metastasis. *F. nucleatum* decreases m6A modifications in CRC cells by suppressing METTL3, promoting CRC metastasis via the YAP pathway. This alteration leads to increased KIF26B expression through decreased m6A modifications, enhancing CRC aggressiveness. Infection by *L. monocytogenes* removes acetyl groups from histone H3K18 in colonic cells, altering gene expression including SMAD1. SIRT2 is activated through its interaction with InlB, leading to modulation of gene expression and immune response. SMAD1's role in CRC metastasis involves promoting the expression of Snail/Ajuba proteins, impacting E-cadherin levels and cell migration. SCFAs produced by the microbiota, predominantly lactobacilli and bifidobacteria, play a significant role in regulating histone modification by influencing the activity of HDACs.

It is postulated that SCFAs may permeate colonic cells via passive diffusion, in conjunction with bicarbonate through counter-transport, or through the utilization of specific transporters such as monocarboxylic acid transporter 1 and sodium-coupled monocarboxylic acid transporter 1 (Ref. 122). Nonetheless, the precise mechanism by which SCFAs impede the aggressiveness and migration of cancerous colonic cells via histone deacetylation remains ambiguous and constitutes a vibrant research avenue. Comparing chromatin maps of colorectal epithelial cells from conventional and germ-free mice, a decrease in histone H3 deacetylation was observed in germ-free mice (Ref. 131). Intriguingly, when SCFAs such as acetate, propionate and butyrate were supplemented, the histone profile of GF mice resembled that of conventional mice. This suggests that these metabolic by-products have the potential to induce changes in histone modifications. Figure 2 illustrates a subset of microbiota and metabolites that modulate pathways associated with epigenetic modifications pertinent to CRC metastasis.

### Microbial impact on non-coding RNA regulation in CRC metastasis

Although research has extensively examined the involvement of non-coding RNAs (ncRNAs) in the development and progression of CRC (Ref. 132), there remains a scarcity of studies that specifically investigate the role of these non-coding transcripts in mCRC. Recently, overexpression of long non-coding RNA (lncRNAs) RAMS11 was found to promote metastasis in CRC patients and its expression correlated with poor disease-free survival (Ref. 133). In separate research, it was found that miR-338-5p plays a role in promoting the migration, invasion and metastasis of CRC by influencing the autophagy pathway associated with PIK3C3 (Ref. 134). Additionally, the ratio between miR-338-5p and PIK3C3 has the potential to serve as a prognostic biomarker for patients with mCRC. Scientists are currently investigating the potential influence of the gut microbiota on the expression of ncRNAs in the host and how this could impact metastasis in CRC. Numerous studies have uncovered that specific gut pathobionts and their metabolites can influence the advancement of CRC by modulating the expression of lncRNAs (Refs 48, 135, 136). For example, infection with *Salmonella typhimurium* has been proven to elevate the expression of LINC00152, an lncRNA that is highly expressed in clinical CRC tumour samples (Ref. 135). This specific lncRNA is associated with cancer cell migration and tumour invasion, ultimately leading to the promotion of CRC metastasis (Ref. 137). *F. nucleatum*, an important gut pathobiont, has been observed to significantly increase the expression of lncRNAs called Keratin7-antisense (KRT7-AS) and Keratin7 (KRT7) in CRC cells (Ref. 48). Notably, the depletion of KRT7-AS resulted in the attenuation of *F. nucleatum*-induced metastasis in CRC. Additionally, it was discovered that KRT7-AS enhanced the migration of CRC cells by upregulating KRT7. Subsequently, the researchers identified the involvement of the NF-*κ*B signalling pathway in the upregulation of KRT7-AS following *F. nucleatum* infection. Consequently, the infection of *F. nucleatum* could activate the NF-*κ*B pathway, leading to the upregulation of KRT7-AS/KRT7, thereby promoting the tumour migration and metastasis of CRC cells. Recent research findings have shown that *F. nucleatum* is capable of inducing an upregulation in the expression of lncRNA EVADR, leading to an increase in the metastatic potential of CRC cells both *in vivo* and *in vitro* (Ref. 138). The underlying mechanism involves the elevated levels of EVADR acting as a versatile scaffold for the Y-box binding protein 1 (YBX1), thus facilitating the direct enhancement of the translation of epithelial–mesenchymal transition (EMT)-related factors such as Snail, Slug and Zeb1. These research findings indicate that the *F. nucleatum*-induced EVADR plays a crucial role in promoting CRC metastasis through YBX1-mediated translation. Furthermore, a direct correlation has been established between an overabundance of *F. nucleatum* and upregulation of miR-21 in CRC (Ref. 139). Mechanistically, experimental evidence has confirmed that the elevated miR-21 level leads to the induction of invasion, intravasation and metastasis in CRC by suppressing the expression of the Pdcd4 protein (Ref. 138). The previous work conducted by Bao *et al*. has provided insight into the role of *B. fragilis*-associated lncRNA1 (BFAL1) in CRC tissues and its involvement in ETBF carcinogenesis (Ref. 140). The researchers observed a significant upregulation of BFAL1 in CRC cells treated with ETBF. Functionally, it was discovered that ETBF contributes to tumour growth by activating the Ras homologue, which is linked to the MTORC1 binding/mammalian target of rapamycin (RHEB/mTOR) pathway through BFAL1. Moreover, BFAL1 was found to regulate the expression of RHEB by competitively interacting with microRNAs miR-155-5p and miR-200a-3p, thereby fostering vascular metastasis in CRC. From a clinical perspective, both elevated BFAL1 expression and the presence of abundant ETBF in CRC tissues were associated with unfavourable prognoses among patients with CRC.

**Figure 3.**
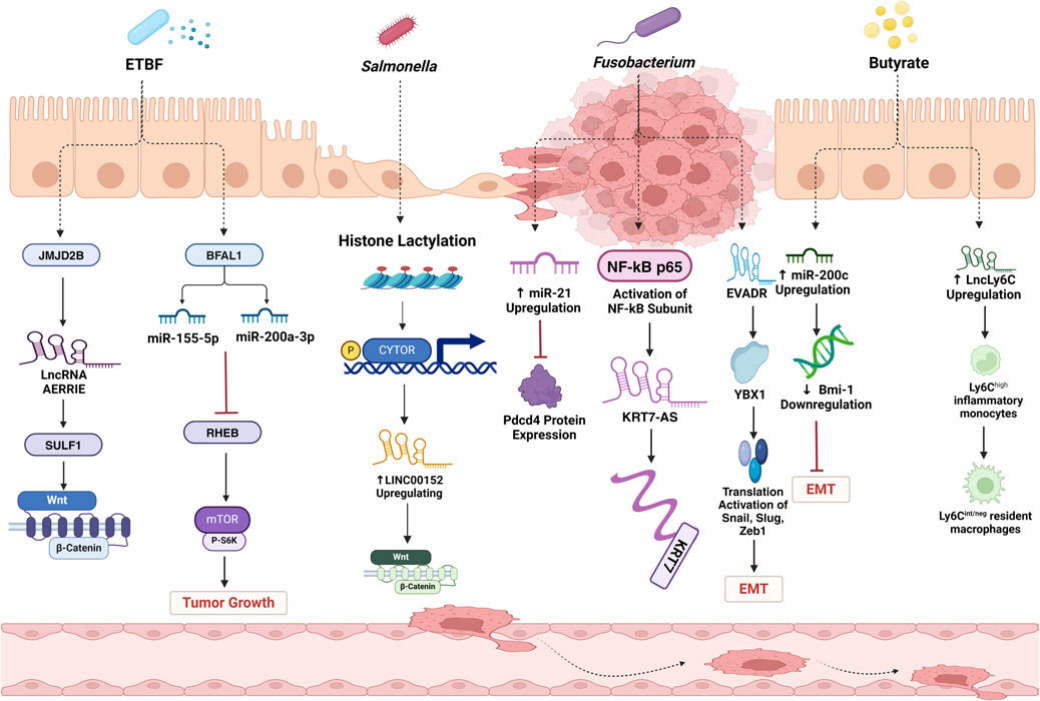
A subset of microbiota and metabolites that influence pathways linked to the aberrant regulation of non-coding RNAs relevant to colorectal cancer metastasis. Gut microbiota, like *Salmonella* spp., *F. nucleatum* and *B. fragilis*, can modulate the expression of ncRNAs such as LINC00152, KRT7-AS, EVADR and AERRIE leading to increased metastasis in CRC. Butyrate from the microbiota can influence the expression of lncLy6C, miR-203 and miR-200c, impacting crucial pathways involved in CRC metastasis.

Moreover, the butyrate originating from the gut microbiota possesses the capability to upregulate the expression of the lncLy6C. It has been experimentally validated that butyrate-induced lncLy6C plays a significant role in inducing the conversion of Ly6C^high^ pro-inflammatory macrophages to Ly6C^low/neg^ resident macrophages, in collaboration with transcription factor C/EBP*β*-mediated Nr4A1 (Ref. 136). Ly6C^high^ monocytes, recognized as a subtype of tumour-associated macrophages, exert a significant influence as promoters of metastasis within the tumour microenvironment. They actively participate in orchestrating the majority, if not all, of the five pivotal steps involved in tumour metastasis (Ref. 141). Butyrate has also been discovered to upregulate the expression of miR-203, thereby inhibiting clone formation, proliferation and invasion, and promoting apoptosis in CRC cells (Ref. 142). This effect is accomplished by downregulating neural precursor cell expressed developmentally down-regulated gene 9 (NEDD9) which is a member of the non-catalytic scaffolding proteins family and a biomarker associated with adverse prognosis, heightened metastatic capacity and chemotherapy resistance (Ref. 142). Moreover, the upregulation of NEDD9 triggers the activation of the Wnt/ß-catenin signalling pathway in CRC. Additionally, NEDD9 facilitates metastatic activity by promoting the internalization of E-cadherin from the cellular membrane and its subsequent degradation in lysosomes (Ref. 143). Furthermore, butyrate plays a crucial role in suppressing the migration of CRC cells by upregulating the expression of miR-200c (Ref. 144). This, in turn, results in a decrease in the levels of BMI1, a gene closely associated with crucial processes such as EMT, invasion of cancer stem cells, cell migration, metastasis and resistance to chemotherapy. Table 2 outlines the potential influence of the gut microbiota and their related metabolic processes on the aggressiveness and spread of CRC through the perturbation of non-coding RNA regulation. Figure 3 depicts a subset of microbiota and metabolites that influence pathways linked to the dysregulation of non-coding RNAs relevant to colorectal cancer metastasis.

**Table 2.** A summary of miRNAs implicated in CRC metastasis, highlighting their functions in CRC metastasis and key references

Microbe/Microbial factor(s)	miRNA	miRNA function in CRC	miRNA target genes	Regulatory mechanism	Interaction with signalling pathways	Clinical implications	References
Gut dysbiosis	Up-regulation of miR-31	Associated with distant metastasis	SATB2; ITGA5	Modulation of adhesion molecules	Wnt signalling pathway; EMT regulation	Indicates metastatic potential	(Ref. 152)
Overrepresentation of *F. nucleatum*	Up-regulation of miR-21; miR-1246; miR-92; and miR-27a	Promotes invasion and metastasis	PTEN; PDCD4	Inhibition of tumour suppressors	Akt; MAPK/ERK pathways	Associated with poor prognosis	(Refs 138, 139)
Overrepresentation of *E. coli*	miR-20a	Promotes invasion and metastasis	NR4A3	Inhibition of tumour suppressors	MMP-9 and E-cadherin pathways	Indicates metastatic potential	(Ref. 153)
Overrepresentation of Bacteroidetes phylum	Down-regulation of miR-141	Suppresses metastasis	ZEB1; SIP1	Inhibition of EMT	TGF-*β*/Smad pathway; Maintenance of epithelial phenotype	Potential therapeutic target	(Ref. 154)
Overrepresentation of ETBF	Attenuation of miR-200 function	Inhibits epithelial–mesenchymal transition and metastasis	ZEB1; ZEB2	Inhibition of EMT	Wnt/*β*-catenin pathway; Maintenance of epithelial phenotype	Associated with better prognosis	(Ref. 140)
Depletion of *F. prausnitzii*	Down-regulation of miR-203	Suppresses tumour invasion	EIF5A2	Inhibition of EMT	PI3 K/Akt; *β*-catenin pathways	Indicates metastatic potential	(Ref. 155)
Microbial-derived butyrate	Down-regulation of miR-92a	Enhances CRC cell migration	FBXW7; PTEN	Promotion of cell motility	PI3 K/Akt pathway, E-cadherin downregulation	Correlated with advanced stages	(Ref. 156)

Abbreviations: CRC, colorectal cancer; ETBF, enterotoxigenic *Bacteroides fragilis*; EMT, epithelial-to-mesenchymal transition.

## Microbial modulation of gut microbiome: a new frontier in personalized cancer therapy and early diagnosis of mCRC

Increasing evidence has consistently demonstrated a notable association between the effectiveness of immunotherapy and the specific composition of the gut microbiome in individuals diagnosed with metastatic malignancies. The evolving understanding of the influence of gut microbiota on metastasis-related pathways holds significant implications for personalized cancer therapy. Recent investigations have explored the practical implications of this knowledge, highlighting how interventions targeting the modulation of the gut microbiome could be utilized to personalize therapeutic approaches by affecting metastasis-related pathways (Ref. 145). In the realm of immunotherapeutic strategies, antibiotic treatment has emerged as a viable option due to its ability to deplete the gut microbiome, triggering an antitumour immune response and impeding tumour growth in metastatic mouse models. Previous studies have highlighted the capacity of antibiotic treatment to diminish bacterial presence within CRC tumours, consequently curtailing tumour invasion and metastasis. Efforts have been made by researchers to harness the potential of probiotics, prebiotics and synbiotics as adjuvant therapies for the prevention and management of metastatic CRC. Probiotics have exhibited the ability to repress various cell types and pathways implicated in the metastatic cascade, modulate gene expression related to cell transformation, migration and invasion, as well as influence the immune system dynamics through the modulation of T-regulatory cell differentiation and anti-inflammatory cytokine production, which collectively contribute to metastasis suppression (Ref. 146). Furthermore, microbial metabolites like short-chain fatty acids have demonstrated inhibitory effects on the invasion, adhesion and metastasis of colorectal tumour cells (Ref. 147). Notably, butyric acid has shown efficacy in mitigating tumour cell apoptosis, diminishing cancer cell metastasis and counteracting carcinogens by upregulating detoxification enzyme expression (Ref. 148). Moreover, compelling evidence suggests that probiotics endowed with metabolic activity can potentially alter the chemical composition of chemotherapy agents, thereby impacting their pharmacological activity, local concentration and overall effectiveness. The utilization of microbial biomarkers in the development of diagnostic tests for the initial diagnosis of CRC holds great potential in enabling early detection of mCRC. Previous investigations have explored the viability of faecal microbiota as a screening tool in various clinical groups, including healthy individuals, those with adenoma, and those with carcinoma, for the early detection of CRC (Ref. 16). However, these studies are considered preliminary, and more in vitro and in vivo studies incorporating robust confirmatory tests for each specific disease are necessary to establish a comprehensive microbiome signature. Moreover, comprehending the interplay between intestinal bacteria, their metabolites, the immune system and tumour microenvironments, as well as understanding the association between microbial populations and the response to antitumour therapies, can contribute to the effective treatment of mCRC. Notably, the selective reduction of bacterial taxa, facilitated by approaches such as antibiotic exposure or other stressors, has been observed to diminish immunotherapy responses. Furthermore, the presence of specific microorganisms in other areas of the body can potentially interfere with treatment outcomes. For instance, gut pathobionts metabolizing and deactivating the active form of anticancer drugs can dampen the efficacy of chemotherapy, adversely affecting tumour responses (Ref. 149). Accordingly, the presence of particular strains may possess the ability to modulate cancer progression and therapeutics. Such insights not only increase the prospects of precision medicine pertaining to the gut microbiota in terms of treatment and prognosis but also position the microbiota as a next-generation therapeutic avenue with a potentially transformative role in this area.

## Conclusion

Available evidence suggests that the microbial community and its metabolites may play a role in the development and progression of mCRC. Mechanisms such as epigenetic alterations and changes in gene expression have provided insights into the complex relationship between the human microbiome and metastasis in CRC. However, these theories do not fully explain the involvement of systemic factors, including lifestyle, diet and ageing, in the interaction between cancer-related mechanisms. The gut microbiota, as one of the earliest and most significant environmental factors encountered in the human body, could potentially modulate various cellular signalling pathways and promote tumour metastasis, as previously mentioned. This could account for the impact of systemic factors on both cancer development and pathways related to tumour invasion. Nevertheless, further efforts are required to uncover the specific mechanisms by which systemic factors, such as the gut microbiota, regulate the processes of cancer initiation and progression.

## Data Availability

All data generated or analysed during this study are included in this published article.
